# Transient Receptor Potential channels: A Global Bibliometric analysis From 2012 to 2021

**DOI:** 10.1080/19336950.2021.1983100

**Published:** 2021-11-13

**Authors:** Xueping Zhu, Chuanxi Tian, Yan Zhou, Jingjing Shi, Guozhen Yuan, Limei Zhang, Yuchen Jiang, Wenjing Xue, Yihang Du, Yuanhui Hu

**Affiliations:** a Department of Cardiology, Guanganmen Hospital, China Academy of Chinese Medical Sciences, Beijing, China; b Clinical Graduate Department, Graduate School of Beijing University of Chinese Medicine, Beijing, China; c Department of Traditional Chinese Medicine for Pulmonary Diseases,China-Japan Friendship Hospital, Beijing, China

## Introduction

The transient receptor potential (TRP) channels, nonselective ion channels, mediate the fluxes of various types of cations across the cell membrane such as Na+, K+, Mg2+, and Ca2 + . TRPA (Ankyrin), TRPC (Canonical), TRPM (Melastatin), TRPV (Vanilloid), TRPP (Polycystin), and TRPML (Mucolipin) are TRP major families members.

These channels play essential roles in diverse physiologic processes, and participate in virtually every sensory modality. TRPs can be activated by chemicals, temperature, stretch/pressure, osmolarity, pH, and so on, and play a major role in the five primary senses, such as vision, taste, hearing, smell, and touch. In recent years, TRP channels are widely studied in the field of nervous, intestinal, renal, urogenital, respiratory, and cardiovascular systems in diverse therapeutic areas including pain and itch, headache, pulmonary function, oncology, neurology, visceral organs, and genetic diseases [1].

Bibliometric analysis has been widely used to calculate the productivity of countries, institutions, authors, and the frequency of keywords to explore research hotspots/frontiers in specific fields [2–4]. In the present study, we performed a bibliometric analysis to systematically evaluate the TRP channels studies from 2012 to 2021 by CiteSpace and VOSviewer to provide researchers with some direction regarding TRP channels research [5,6].

## Data source and search

**Figure 1.**
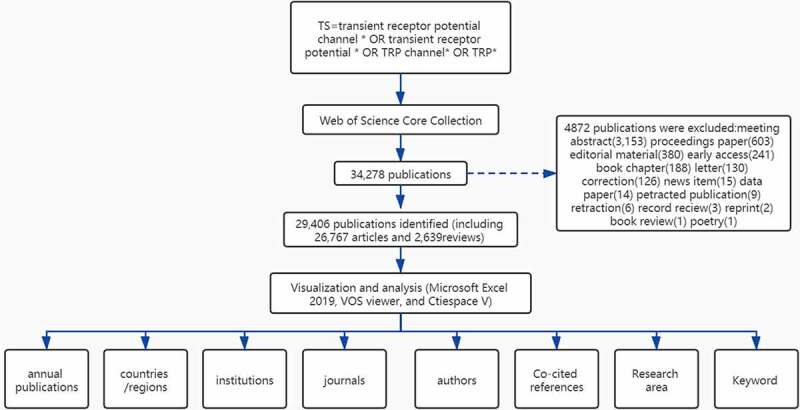
Flow chart for the analysis of TRP channels researches

The publications were obtained from the Core Collection database of Web of Science (WoS) (http://apps.webofknowledge.com) which is considered the most prominent database of scientific publications on many research topics. The data search was conducted on 10 July 2021. The strategy used during the search was [TS = transient receptor potential channel * OR transient receptor potential * OR TRP channel* OR TRP*] AND [Language = (English)] AND [Year Range = (2012–2021)]. 34,278 publications were obtained, and the following documents were excluded: meeting abstract(3,153) proceedings paper(603) editorial material(380) early access(241) book chapter(188) letter(130) correction(126) news item(15) data paper(14) petracted publication(9) retraction(6) record reciew(3) reprint(2) book review(1) poetry(1). In total, only 29,406 records (26,767 articles and 2,639 reviews) were analyzed. The data were collected within 1 day to avoid any potential deviation due to the daily updating of the database. The VOSviewer 1.6.16 was used to identify top countries, institutions, authors, and journals. The CiteSpace 5.8. R1 was used to analyze keywords, co-cited references, and trends. The data analysis flow chart is shown in (Figure 1). In this study, the data were downloaded directly from the database as secondary data without further animal experiments. Therefore, no ethical approval was required.

## Annual publication output

**Figure 2.**
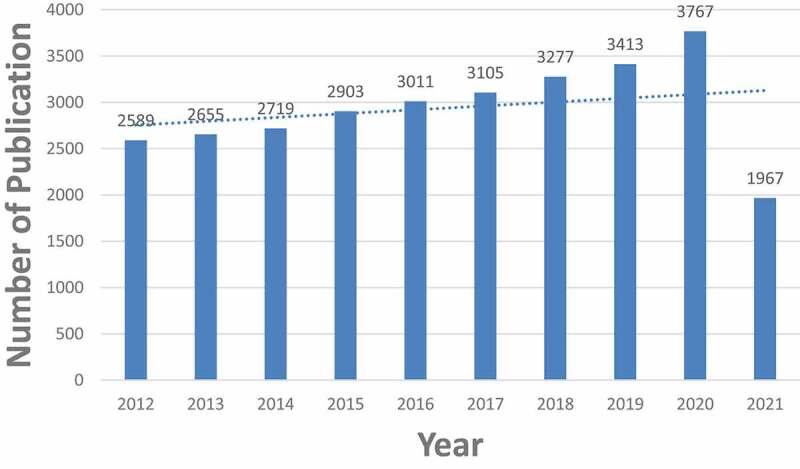
The number of annual publications on TRP channels research from 2012 to 2021

**Figure 3.**
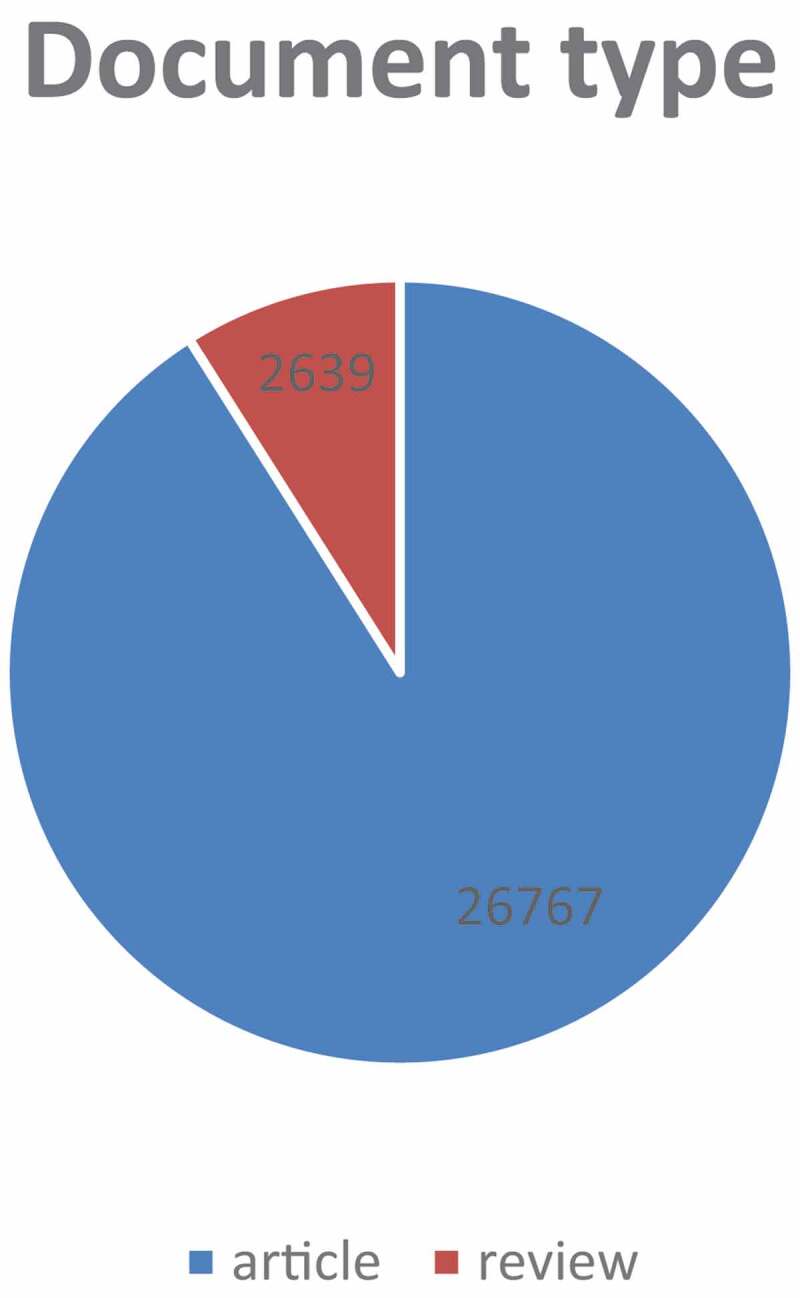
Document type. Blue represents articles and Orange represents reviews

A total of 29,406 TRP-related publications were obtained. To explore the trends in TRP channel research, we showed the number of articles per year in the form of a histogram. Because the annual number of published papers reflects the pace of subject knowledge and is a significant indicator for studying the trends in the field [7]. As shown in (Figure 2), the annual number of relevant publications started increasing rapidly from 2012 to 2021, indicating a steady development and more attention of TRP. And up to 10 July 2021, more than 1,967 literature have been published in 2021. Furthermore, articles account for about 91.03% in terms of document type (Figure 3), which indicates the greater emphasis paid on original studies in the area of TRP.

## Active countries and institutions

**Figure 4.**
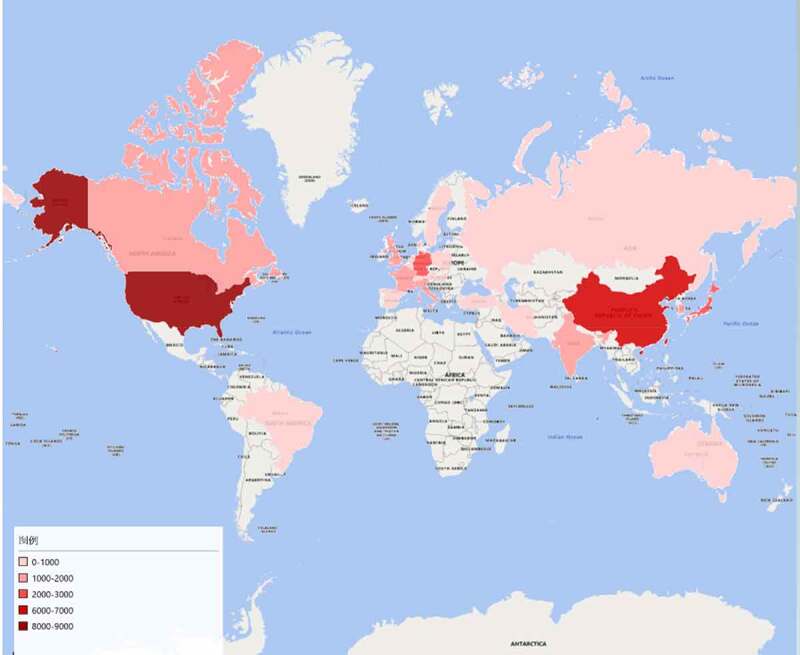
Geographical distribution map of global publications related to TRP channels

**Figure 5.**
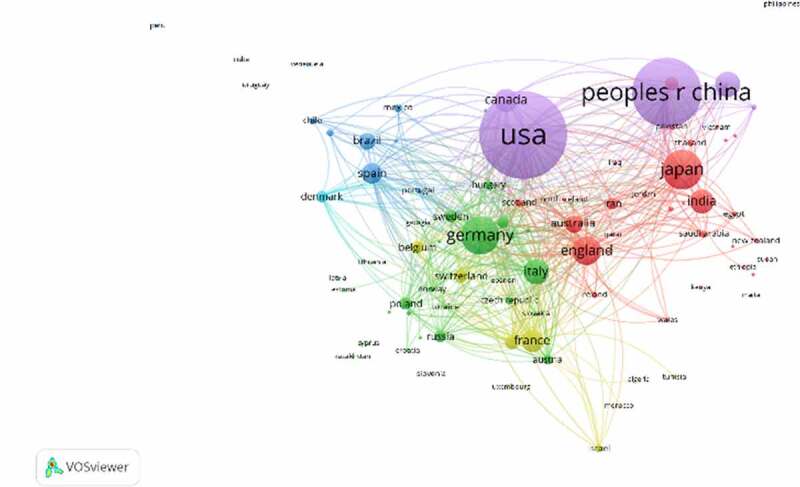
The network of countries/territories engaged in TRP channels research

**Figure 6.**
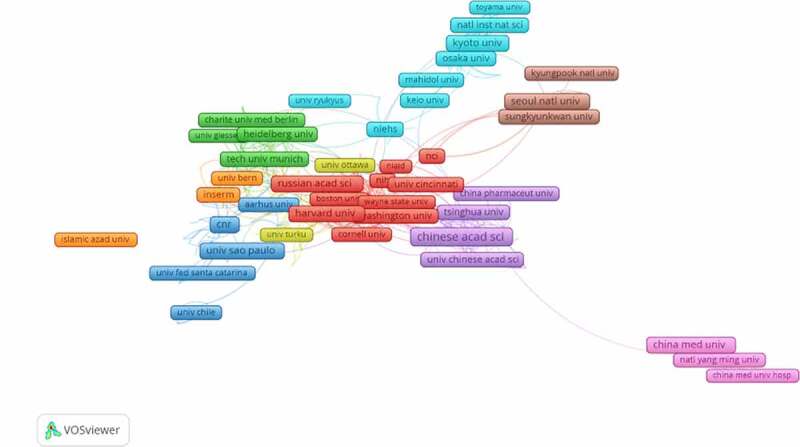
The network of institutions engaged in TRPs channel research

Geographical distribution map of global productivity revealed that articles on TRP channels had been mainly published from North American, Asia, and European countries (Figure 4). (Table 1) lists the top 10 countries and institutions ranked by the numbers of publications on the TRP channels. The 29,406 literature were published by more than 16,103 research institutions in 132 countries/territories. The co-occurrence map provides valuable information on influential research teams and helps researchers to identify the cooperative relationship [8–10]. Countries and institutions co-occurrence maps are shown in (Figures 5) and 6. The leading country was the USA, which took up 30.11% (8853/29,406) of the total number of publications, followed by CHINA (6259, 21.285%) and JAPAN (2706, 9.202%). (Figure 5) shows that the United States attached great importance to cooperation, and had close collaborations with China, Japan, and Germany. The most productive scientific research institution was the Chinese Academy of Sciences, produced the highest number of publications on TRP channels (525), followed by Seoul National University (284). As shown in (Figure 6), the collaboration map had 500 nodes and 12,343 links. The 500 institutions formed nine clusters with different colors. The co-occurrence map of institutions showed that scientific cooperation among institutions was greatly affected by the geographical location, and there are more cooperations among institutions in the same region (Figure 6).

**Table 1.** The top 10 countries and institutions contributed to publications on TRP channels research

Rank	Country/ Territory	Frequency	% of 29,406	Institution	Frequency	% of 29,406
1	USA	8853	30.106	Chinese Academy of Sciences	525	1.785
2	PEOPLES R CHINA	6259	21.285	Seoul National University	284	0.966
3	JAPAN	2706	9.202	Kyoto University	267	0.908
4	GERMANY	2491	8.471	Shanghai Jiao Tong University	223	0.758
5	ENGLAND	1646	5.597	Zhejiang University	221	0.752
6	ITALY	1352	4.598	Harvard University	220	0.748
7	SOUTH KOREA	1349	4.587	Russian Academy of Sciences	219	0.745
8	INDIA	1257	4.275	Johns Hopkins University	216	0.735
9	CANADA	1225	4.166	Univ Illinois	213	0.724
10	FRANCE	1137	3.867	Peking University	212	0.721

## Active journals

The 29,406 literature were published in 3,992 journals. (Table 2) lists the top 10 journals that published articles on TRP channels research. The Plos One had the highest number at 766 (2.61%) (IF2020 = 3.24), followed by Science Reports published 590 papers (2.01%) (IF2020 = 4.379, and the Journal of Biological Chemistry ranked third at 447 articles (1.52%) (IF2020 = 5.157).

**Table 2.** The top 10 journals that published articles on TRP channels research

Rank	Journal	Frequency	% of 29,406	Total Cites	IF 2020	Country Affiliation
1	PLOS ONE	766	2.605	857,723	3.24	United States
2	SCIENTIFIC REPORTS	590	2.006	541,615	4.379	England
3	JOURNAL OF BIOLOGICAL CHEMISTRY	447	1.52	397,453	5.157	United States
4	INTERNATIONAL JOURNAL OF MOLECULAR SCIENCES	410	1.394	139,463	5.923	United States
5	PROCEEDINGS OF THE NATIONAL ACADEMY OF SCIENCES OF THE UNITED STATES OF AMERICA	272	0.925	799,058	11.205	United States
6	JOURNAL OF NEUROSCIENCE	198	0.673	186,015	6.167	United States
7	BIOCHEMICAL AND BIOPHYSICAL RESEARCH COMMUNICATIONS	196	0.667	105,148	3.575	United States
8	BRITISH JOURNAL OF PHARMACOLOGY	193	0.656	42,870	8.739	England
9	CELL CALCIUM	193	0.656	6842	6.817	Scotland
10	BIOCHEMISTRY	180	0.612	76,745	3.162	United States

## Active authors

**Figure 7.**
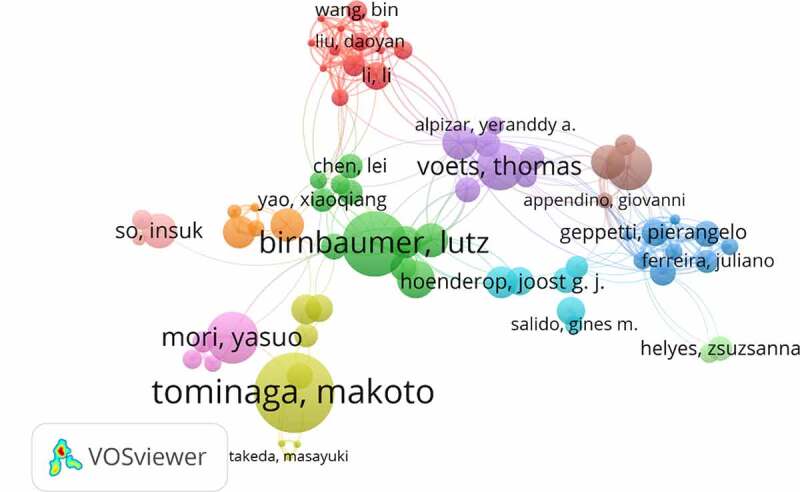
The network of authors contributed to TRP channels research

Author co-occurrence map can provide information on influential research groups and potential collaborators. It can help researchers to find potential collaborators [8,9]. A total of approximately 100,000 authors were obtained in the 29,406 publications elated to TRP channels research. The networks shown in (Figure 7) indicate the cooperation among authors, and the top10 active authors are listed in (Table 3). Tominaga, Makoto, mainly focused on The Role of TRP Channels in Thermosensation and Nociception [11,12], contributed the most papers (118 publications, 0.40%), followed by Birnbaumer, Lutz and Mori, Yasuo with 96 and 76 publications, respectively. There was an active collaboration among the productive authors.

**Table 3.** The top10 active authors in TRP channels research

Rank	Author	Frequency	% of 29,406
1	Tominaga,Makoto	118	0.40%
2	Birnbaumer,Lutz	96	0.33%
3	Mori,Yasuo	76	0.26%
4	Voets,Thomas	70	0.24%
5	Di Marzo,Vincenzo	68	0.23%
6	Gudermann,Thomas	56	0.19%
7	Nilius,Bernd	52	0.18%
8	Freichel,Marc	50	0.17%
9	Wang,Yan	49	0.17%
10	Wang,Jian	48	0.16%

## Co-cited references

**Figure 8.**
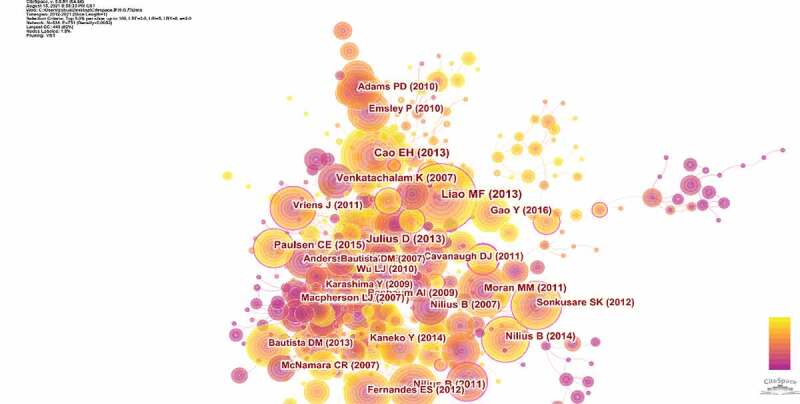
The analysis of Co-cited references: Co-citation network of references from publications on TRP channels research

29,406 publications were visualized and analyzed using CiteSpace 5.8.R1 with a period time from 2012 to 2021, and a time slice of 1 was chosen for the analysis of the co-cited references. The network of co-cited references on TRP channels consists of references with higher centrality and citation counts which is presented in (Figure 8). The highly cited references were analyzed to determine the key knowledge base in the field. The top 10 highest co-cited references are summarized in (Table 4). These articles laid the foundation for studying the structure and mechanism of TRP channels. And these reviews provided a theoretical basis for the study of TRP channels.

**Table 4.** The top10 Co-cited references in TRP channels research

Rank	Frequency	Author	Year	Source	Co-cited Reference
1	476	Maofu Liao	2013	Nature	Structure of the TRPV1 ion channel determined by electron cryo-microscopy
2	418	David Julius	2013	Annu Rev Cell Dev Biol	TRP channels and pain
3	415	Erhu Cao	2013	Nature	TRPV1 structures in distinct conformations reveal activation mechanisms
4	260	Kartik Venkatachalam	2007	Annu Rev Biochem	TRP channels
5	260	Bernd Nilius	2011	Genome Biol	The transient receptor potential family of ion channels
6	249	Candice E Paulsen	2015	Nature	Structure of the TRPA1 ion channel suggests regulatory mechanisms
7	240	Magdalene M Moran	2011	Nat Rev Drug Discov	Transient receptor potential channels as therapeutic targets
8	225	Bernd Nilius	2014	Pharmacol Rev	Transient receptor potential channels as drug targets: from the science of basic research to the art of medicine
9	224	Bernd Nilius	2007	Physiol Rev	Transient receptor potential cation channels in disease
10	217	Yuan Gao	2016	Nature	TRPV1 structures in nanodiscs reveal mechanisms of ligand and lipid action

The first highly co-cited article was “Structure of the TRPV1 ion channel determined by electron cryo-microscopy.” (476 citation rate), in which Maofu Liao explored advances in electron cryo-microscopy to determine the structure of TRPV1, at 3.4 Å resolution and provided a structural blueprint for understanding unique aspects of TRP channel function [13]. There were other three co-cited references published in Nature: In 2013, Erhu Cao revealed that TRPV1 opening was associated with structural rearrangements in the outer pore, and suggested a dual gating mechanism [14]. In 2015, Candice E Paulsen used single-particle electron cryo-microscopy to determine the structure of full-length TRPA1 to ∼4 Å resolution in the presence of pharmacophores, including a potent antagonist [15]. One year later, Yuan Gao demonstrated the power of combining electron cryo-microscopy with lipid nanodisc technology to ascertain the structure of the rat TRPV1 ion channel in a native bilayer environment [16].

## Research area analysis

**Figure 9.**
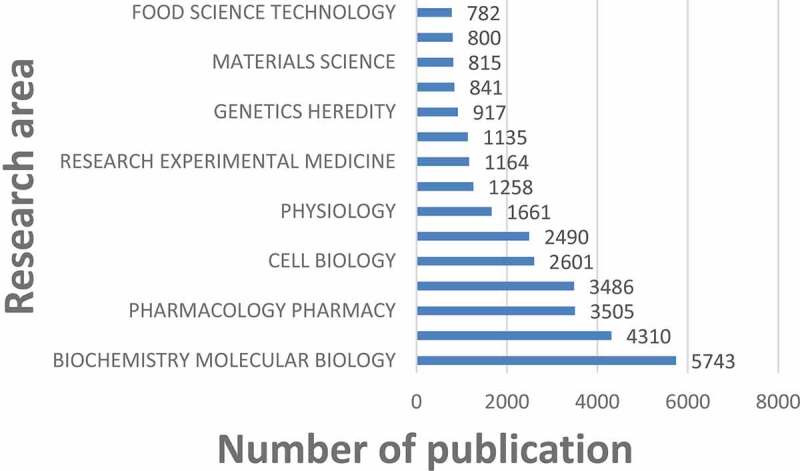
The 15 research areas on TRP channels research

(Figure 9) shows the top 15 research areas that appeared in publications related to TRP channels research from 2012 to 2021. BIOCHEMISTRY MOLECULAR BIOLOGY, CHEMISTRY, and PHARMACOLOGY PHARMACY are the top three areas where TRP channels are more studied.

## Keyword co-occurrence

Keywords were extracted from titles and abstracts of all the 19,406 publications, which is the crucial content of research. Keyword co-occurrence analysis provides a reasonable description of research hotspots, and burst keywords can represent research frontiers over a period of time [17].

**Figure 10.**
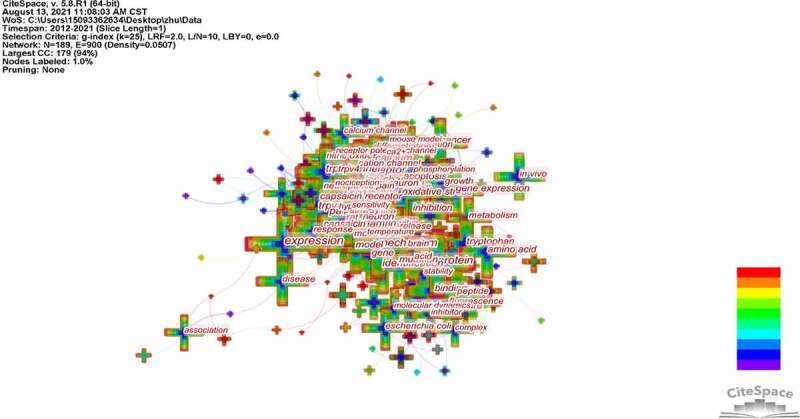
The analysis of keywords inTRP channels research

CiteSpace 5.8.R1 was used to construct an acknowledge map of keyword co-occurrence (Figure 10) and identified the top 20 keywords in TRP channels research from 2012 to 2021(Table 5), according to frequency. The top keywords were “expression,” “activation,” “protein,” “mechanism,” “receptor,” “ion channel,” “trpv1,” “channel,” “pain,” “cell,” “calcium,” “inflammation,” “identification,” “trp channel,” “inhibition,” “tryptophan,” “gene,” “rat,” “binding,” “oxidative stress.” Therefore, research hotspots can be summarized in the following aspects

**Table 5.** Top 20 keywords in terms of frequency in nTRP channels research

Rank	keywords	Frequency	Rank	keywords	Frequency
1	expression	3419	11	calcium	1177
2	activation	3270	12	inflammation	1151
3	protein	2180	13	identification	1128
4	mechanism	2028	14	trp channel	1124
5	receptor	2011	15	inhibition	1062
6	ion channel	1786	16	tryptophan	1036
7	trpv1	1733	17	gene	1005
8	channel	1718	18	rat	965
9	pain	1366	19	binding	929
10	cell	1300	20	oxidative stress	924

### ① TRPV1

TRPV1 channel involves the regulation of many important physiological and pathological processes. In many diseases, the TRPV1 channel may be an effective treatment in the future, such as inflammation, cardiovascular disease, pain, diabetes, schizophrenia, pneumonia, psoriasis, and so on.

*a) TRPV1 and inflammation*: TRPV1 is closely associated with inflammation and is responsible for the neurogenic component of inflammation development, which is large expressed in nociceptive neurons of the peripheral nervous system [18]. Activation of TRPV1 channels can release neuropeptides from nerve endings and simultaneously increase vascular permeability. Research shows that the vascular permeability in the respiratory tract inflammation model increased after microinjection of the TRPV1 agonist and decreased after microinjection of the TRPV1 antagonist [19]. Fibroblasts are originally TRPV1 negative cells, and after stimulating by proinflammatory agents, such as TNF-α, IL-1α, and LPS cells for 24 and 48 hours, fibroblasts begin to synthesize TRPV1 mRNA, which confirming the functionality of the membrane-embedded receptors [20].

*b) TRPV1 and cardiovascular disease*: TRPV1 is highly expressed in the smooth muscles of coronary arterioles, skeletal muscles, adipose tissue, and microvessels (vasa vasorum) [21]. It was found that TRPV causes the Bezold–Jarisch cardiopulmonary chemical reflex manifested in a short-term drop in BP, bradycardia, and apnea [22], and the transient Bezold–Jarisch reflex is mediated by the TRPV1 activation in the sensory neurons, while TRPV1 activation in arterioles induces the BP increase [21]. TRPV1 also affects circulation through the spinal cord neurons and DRG neurons. The experiment shows that on the myocardial ischemia-reperfusion, additional activation of the spinal cord TRPV1 contributed to a severer myocardial injury; however, intrathecal administration of the TRPV1 antagonist capsazepine reduced the size of the infarct area [23].

### ②- TRP channels and pain

Pain can be divided into three categories including nociceptive pain, inflammatory pain, and neuropathic pain. studies have shown that TRP channels have a bi-directional effect on the regulation of crucial pain processes: transduction, transmission, and modulation [24]:

*a) Transduction is the process that nerve endings detect tissue-damaging injury*: TRPs are nonselective cation channels with relatively high Ca2+ permeability, expressed in peripheral and central nervous system (CNS) terminals, and detect/transduce painful signals. For example, TRPV1 can be activated by capsaicin, acid, and heat [25].

*b) transmission is the process that relay signal messages from the site of tissue injury to the central nervous system*: Though the dorsal root ganglion (DRG) and trigeminal ganglion (TG), afferent inputs from nociceptors enter the CNS for transmission to the cerebral cortex for interpretation [26]. TRPV1 channels are expressed in DRG and TG and affect transmission in nociceptive neurons. TRPA1 is expressed in the dorsal horn of the spinal cord and promotes the release process of glutamate [25]. TRPM2 and TRPM8 channels are also expressed in DRG and TG [27].

*c) Modulation is the neural process that reduces the transmission system and pain perception*: It is worth noting that activating some TRP channels, such as TRPV1, TRPA1, and TRPM8, can modulate pain perception [25,28]. TRPM2-mediated infiltration of macrophages and microglia contribute to the pathogenesis of neuropathic pain, and TRPA1-mediated loss of substance P reduces tactile sensitivity in diabetic neuropathy [29,30]. AS TRP channels play a more and more important role in pain, many TRP channels are considered as potential therapeutic targets for pain management [31,32].

### ③ TRP channels accelerates lung inflammation

TRP channels are expressed in the lung endothelium [33–36]. AS a second messenger, they mediate Ca2+ influx and signaling to regulate endothelial permeability, vasodilation, angiogenesis, inflammation, and pathophysiological response [37–40]. TRP heteromers – TRPC1/4, TRPC3/6, and TRPV1/4-play a crucial role in pulmonary inflammation. They may extend openings to accelerate Ca2+ influx in order to promote a CaM/MLCK-signaling-dependent increase in endothelial permeabilIty, and disrupt endothelial barrier function more profoundly [41].

## Keyword co-occurrence and burst

Keywords were identified and analyzed using strong citation bursts (Table 6) to explore research hotspots, frontiers, and emerging trends over time. As shown in (Table 6), the red line indicates the period of time during which the burst keyword appears [42]. The citation burst time of keywords including “molecular docking”(2017–2021, 43.06), “contribute”(2017–2021, 38.07), “cytokine”(2019–2021, 36.2), “autophagy”(2019–2021, 34.25), “risk”(2019–2021, 33.86), “performance”(2018–2021, 26.79), “antioxidant”(2017–2021, 26.3), and “design”(2018–2021,22.09) has continued to 2021, and are still ongoing, reveals that these directions have great potential.

**Table 6.** Top 25 keywords with the strongest citation bursts

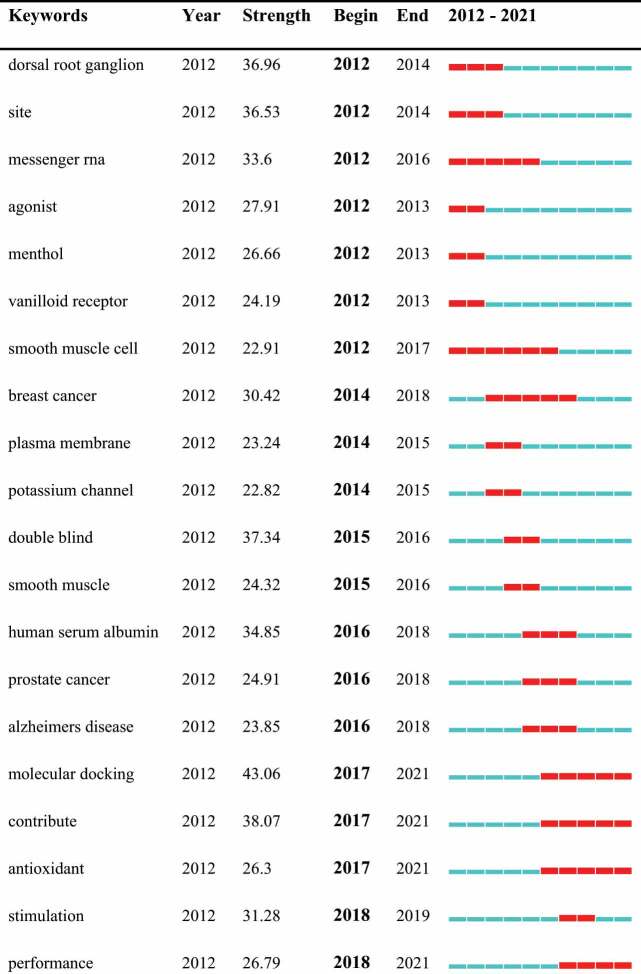

## Conclusions

Based on the WOSCC database, bibliometric and Visual analysis were used to study the characteristics of TRP channels research results from 2012 to 2021. The number of publications on TRP channels has maintained over 2,500 per year. There will be a dramatically increasing number of publications on TRP research based on the current global trends. The hot spots of TRP channels research were “pain,” “calcium,” “inflammation,” and “oxidative stress.” The top research frontiers were “molecular docking,” “cytokine,” and “autophagy.” Bibliometric analysis of the literature on the TRP channels contributes researchers to identify cooperations, find research hotspots, and predict the frontiers of chloride channel research.
